# Antiproliferative and Apoptotic Effects of a Specific Antiprostate Stem Cell Single Chain Antibody on Human Prostate Cancer Cells

**DOI:** 10.1155/2013/839831

**Published:** 2013-12-10

**Authors:** Foroogh Nejatollahi, Soghra Abdi, Mahdi Asgharpour

**Affiliations:** ^1^Recombinant Antibody Laboratory, Department of Immunology, Shiraz University of Medical Sciences, Shiraz 71348-45794, Iran; ^2^Shiraz HIV/AIDS Research Center, Shiraz University of Medical Sciences, Shiraz 71348-45794, Iran

## Abstract

Prostate stem cell antigen (PSCA) is a highly glycosylated cell surface protein which is overexpressed in several malignancies including prostate, pancreas, and urinary bladder cancers. Tumor suppression has been reported by anti-PSCA antibody. Small and high affinity single chain antibodies (scFv) have been introduced as effective agents for cancer immunotargeting approaches. In the present study, we used a phage antibody display library of scFv and selected two antibodies against two immunodominant epitopes of PSCA by panning process. The reactivity of the scFvs for the corresponding epitopes was determined by phage ELISA. The binding specificity of antibodies to PSCA-expressing prostate cancer cell line, DU-145, was analyzed by flow cytometry. The antiproliferative and apoptotic induction effects were evaluated by MTT and Annexin-V assays, respectively. Results represented functional scFv C5-II which could bind specifically to DU-145 cells and significantly inhibited the proliferation of these cells (61%) with no effect on PSCA-negative cells. The antibody also induced apoptosis in the PSCA expressing cells. The percentage of the apoptotic cells after 24 hrs of exposure to 500 scFv/cell was 33.80%. These results demonstrate that the functional anti-PSCA scFv C5-II has the potential to be considered as a new agent for targeted therapy of prostate cancer.

## 1. Introduction

Prostate stem cell antigen is a cell surface antigen belonging to the Thy-1/Ly-6 family of glycosylphosphatidylinositol (GPI) anchored proteins [1]. PSCA expression in normal tissues has shown to be predominantly prostate specific. However, less expression of PSCA has also been detected in other normal tissues including placenta, stomach, and kidney [2]. Elevated levels of PSCA have been reported in over 80% of prostate cancer specimens and in all cases of bone metastasis from prostate cancer patients [3]. The overexpression of PSCA has also been reported in most bladder and pancreatic cancers [4–6]. In the cases of prostate cancer, high levels of PSCA expression have widely been correlated with high Gleason score, advanced tumor stage, seminal vesicle involvement, progression to androgen-independent disease, and bone metastasis [7–10]. Although the role of PSCA in intercellular signaling has been shown, little is known about the regulatory mechanism or biological functions of PSCA [11, 12]. It has been suggested that PSCA could act as both tumor suppressor and tumor promoting antigen based on tumor type, the microenvironment of the tumor, and the crosstalk between PSCA and other molecules [12]. Preclinical data have suggested PSCA as a potential target antigen for both diagnostic and therapeutic applications. Blocking of PSCA with monoclonal antibodies in some mouse models of prostate and pancreas cancers has resulted in the inhibition of tumor growth and prevention of metastasis [13–15].

Recombinant antibodies have recently shown great promise in the replacement of monoclonal antibodies in different medical areas such as immunotherapy against human malignancies [16–19]. Single chain fragment variable (scFv) antibodies are one of the most popular formats of the recombinant antibodies [20]. Advantages of scFvs over the intact antibodies including smaller size, fast penetration and tight binding to target tissue, fast clearance from the body, and better pharmacokinetic properties as well as fully human origin have offered scFvs as desirable tools for both the imaging and therapeutic purposes [21–24].

In the present study, we isolated specific scFv antibodies against immunodominant epitopes of PSCA and evaluated their inhibitory effects on PSCA-expressing cancer cells using cell proliferation and Annexin-V assays.

## 2. Materials and Methods

### 2.1. Selection of Anti-PSCA scFv

A phage antibody display library of scFv was developed as described previously [25, 26]. The library was phage-rescued using M13KO7 helper phage and the specific scFv antibodies were isolated by panning process. Briefly, peptides as epitopes (amino acids 50–64 and 67–81 of PSCA) were coated overnight on immunotubes (Nunc, Roskilde, Denmark). The phage-rescued supernatant (10^10^ PFU/mL) diluted with blocking solution was added to the tubes and incubated for 1 h at room temperature. After adding the log phase TG1 *E. coli* bacteria, the bacterial pellet was grown on 2TY-ampicillin agar plates. Four rounds of panning were performed to isolate specific antibodies against the epitopes. PCR was performed on the clones obtained after panning to investigate the presence of the desired band corresponding to the scFv insert and DNA fingerprinting with Mva-I restriction enzyme revealed the common patterns. One of the clones with the most frequent pattern was selected against each epitope and phage-rescued for further evaluations.

### 2.2. Measurement of scFv Concentration

Concentrations of the selected scFvs were measured using phage concentration determination. The selected phage-rescued supernatant (10 *μ*L) was added to 1 mL TG1 *E. coli* in a logarithmic growth phase and incubated with shaking for 1 h at 37°C. Serially diluted cultures were plated onto 2TY-ampicillin agar plates. Single-chain Fv concentration was then calculated by counting the number of colonies per dilution.

### 2.3. Phage ELISA

Peptides (100 *μ*g/mL) were coated on microtiter 96 well ELISA plate overnight at 4°C (an unrelated peptide was also coated as a negative antigen control). Wells were blocked by blocking solution using 2% skimmed milk in PBS and incubated at 37°C for 2 hrs. After washing with PBS/Tween 20 and PBS, the phage-rescued supernatant containing scFv was added to the plate and incubated at room temperature (RT) for 2 hrs (M13KO7 helper phage was added to peptide coated wells as a negative antibody control). Following washing, anti-fd bacteriophage antibody (Sigma, UK) was added and incubated for 1.5 h. The plate was washed to remove unbounded antibodies and HRP-conjugated anti-fd antibody was added. After 1 h of incubation at RT, the plate was washed and 150 *μ*L of the substrate (1 *μ*L H_2_O_2_ with 0.5 mg/mL ABTS in citrate buffer) was added. The absorbances were detected at 405 nm after 30 mins using an ELISA reader.

### 2.4. Cell Culture

Human prostate cancer cell lines, DU-145 and LNCaP, were purchased from National Cell Bank of Iran (NCBI, Pasteur Institute of Iran, Tehran, Iran). The cell lines were cultured and maintained in RPMI-1640 medium (PAA, Grand Island, NY, USA) and supplemented with 10% fetal bovine serum (PAA), 100 Unit/mL penicillin, and 100 *μ*g/mL streptomycin (BioSera, UK). Cells were grown at 37°C in a CO_2_ incubator.

### 2.5. Flow Cytometry

To examine the specific binding of the selected scFv antibodies to the PSCA on the surface of the cells, flow cytometry was performed. Cells (10^6^) were transferred to flow cytometry tubes. The anti-PSCA scFv antibody (500 scFv/cell) was added to the tubes and incubated for 2 h at 4°C. The M13KO7 helper phage was used as the isotype control. Anti-fd antibody (Sigma, Chemical Co., UK) was added and incubated at RT for 1 h. Following washing, FITC-conjugated anti-fd antibody (Sigma, Chemical Co., UK) was added to the tubes and incubated at RT for 30 min in a dark place. Data were acquired using FACSCalibur (Becton Dickinson, USA).

### 2.6. Cell Proliferation Assay

Cells were seeded onto a 96-well flat-bottomed plate (NUNC, Denmark) at a density of 10^4^ cells/well and incubated overnight at 37°C. DU-145 and LNCaP cell lines were then treated in 6 replicates with various concentrations of scFv (200, 500, and 1000 scFv/cell) for 24 hrs at 37°C. M13KO7 helper phage and 2TY broth media were used as negative controls. MTT reagent (150 *μ*L of 0.5 mg/mL) was added followed by 4 hrs of incubation at 37°C. After purple formazan crystal formation, the supernatant was gently removed and crystal products were solubilized and incubated in DMSO (Merck, Germany) at room temperature overnight. Colorimetric evaluation was performed at 570 nm using a spectrophotometer. The percentage of cell growth was calculated from the absorbance values of untreated and treated cells as follows:

 %  cell  growth = (OD_570_  treated/OD_570_  untreated)∗100.

### 2.7. Annexin-V Assay

Annexin-V/PI assay was carried out to evaluate the possible apoptotic effect of C5-I and C5-II scFv antibodies on PSCA-positive DU-145 cells. For each condition, 10^6^ cells/well were transferred to 6 cm culture plates and incubated overnight at 37°C. Cells were then treated with C5-I and C5-II scFv antibodies (500 scFv/cell) for 24 h at 37°C. DMSO (10% in CM10 for 3 h) was used as positive control. Cells were gently harvested and transferred to flow cytometry tubes, washed with cold PBS, and stained with 2 *μ*L Annexin-V and 2 *μ*L propidium iodide in 100 *μ*L incubation buffer (Roche Applied Science, Germany) for 15 min at RT in the dark and then analyzed by FACSCalibur (Becton Dickinson, Franklin Lakes, NJ, USA).

### 2.8. Statistical Analyses

Data were analyzed using the Mann-Whitney *U* test to compare the means of percentages of cell growth between antibody-treated and untreated cells. All data are presented as the mean ± standard deviation. *P*  value <0.05 was considered statistically significant.

## 3. Results

### 3.1. Anti-PSCA scFv Antibodies

**Figure 1:**
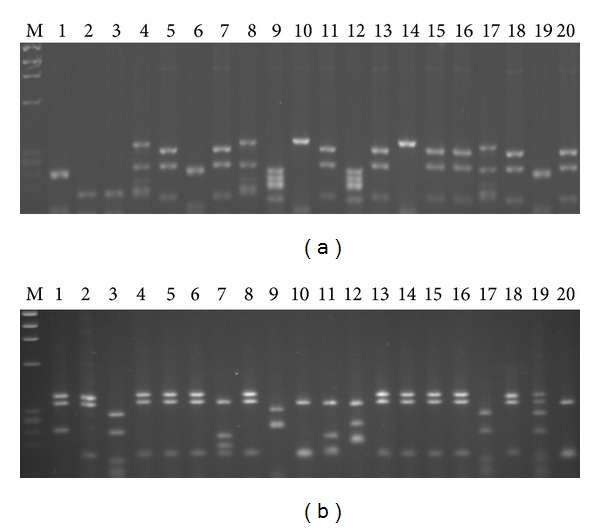
DNA fingerprinting patterns of the clones after panning against peptides I and II (a) and (b). A common pattern with frequency of 40% was obtained for peptide I (lanes 5, 7, 11, 13, 15, 16, 18, and 20) (a) and the common pattern against peptide II showed frequency of 55% (lanes 1, 2, 4, 5, 6, 8, 13, 14, 15, 16, and 18) (b).

Figures 1(a) and 1(b) show DNA fingerprinting of 20 panned clones against the epitopes I and II. Common patterns with the frequencies of 40% (8/20) and 55% (11/20) were obtained against peptides I and II, respectively. One colony from each pattern (clones C5-I and C5-II) was used for further investigations.

### 3.2. Phage ELISA

**Figure 2:**
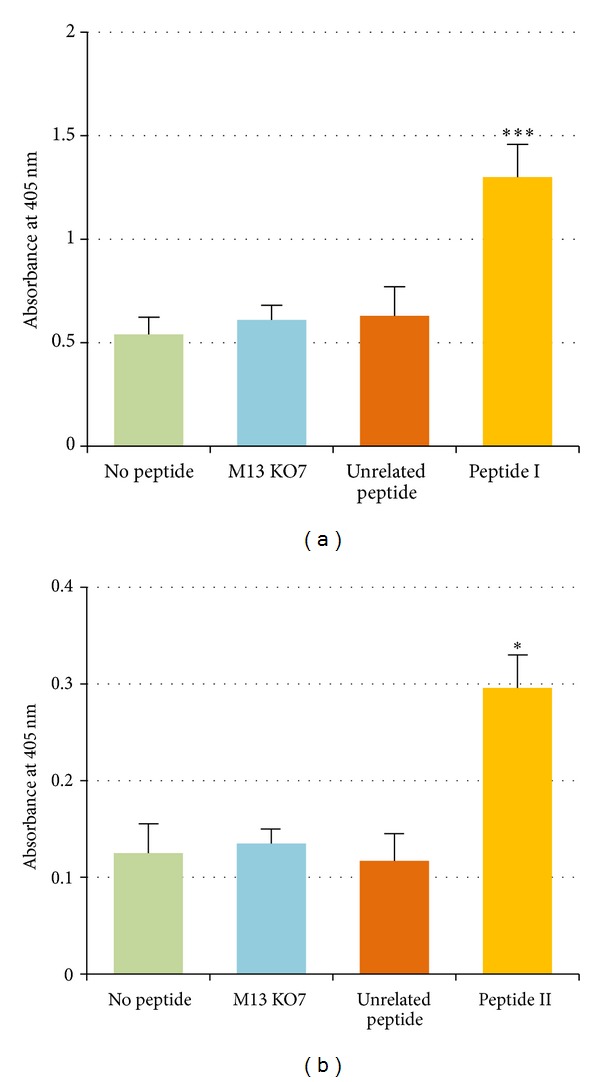
Phage ELISA results of scFv C5-I and scFv C5-II against peptides I (a) and II (b). The both antibodies bound to the corresponding peptides significantly higher than the wells with no peptide (*P value <0.05). No reactivity was detected for unrelated peptide with the scFvs and M13KO7 helper phage with the peptides.

Phage ELISA was performed to determine the binding specificity of selected scFvs to the corresponding peptides. As shown in Figure 2, C5-I and C5-II scFvs bound to peptides I and II of PSCA, respectively. The absorbances were significantly higher than the wells with no peptide (*P*  value <0.05). The helper phage M13KO7 showed no reactivity to the epitopes.

### 3.3. Flow Cytometry

**Figure 3:**
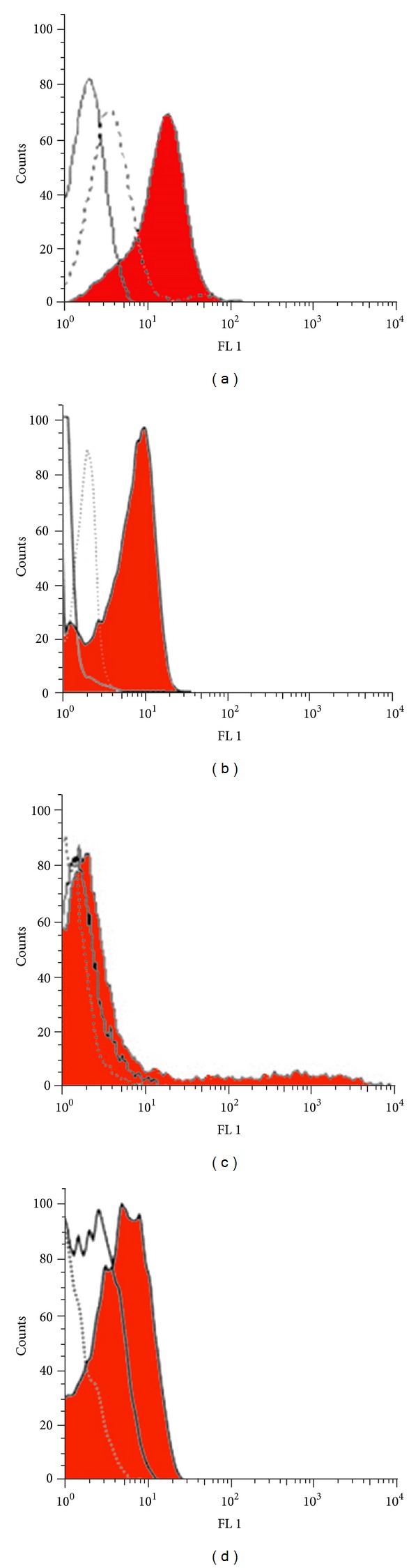
Flow cytometry analysis of scFv C5-I (a) and scFv C5-II (b) binding to PSCA-expressing DU-145 cells and PSCA-negative LNCaP cells (c) and (d). The both scFv antibodies bind to PSCA-expressing cell line as shown by the shift in fluorescence intensity value with the DU-145 cells compared to intensity measured from the isotype control (a) and (b). The fluorescence intensity measured from LNCaP cells was nearly identical to the values measured from isotype control or untreated cells (c) and (d). Filled area represents treated cells, white area represents untreated cells and dotted line represents isotype (M13KO7) treated cells.

Flow cytometry revealed the binding ability of the C5-I and C5-II scFvs to PSCA on the surface of DU-145 cells. The results showed that both antibodies bound to DU-145 cells specifically in comparison to PSCA-negative cell line, LNCaP (Figure 3).

### 3.4. Cell Proliferation (MTT) Assay

**Figure 4:**
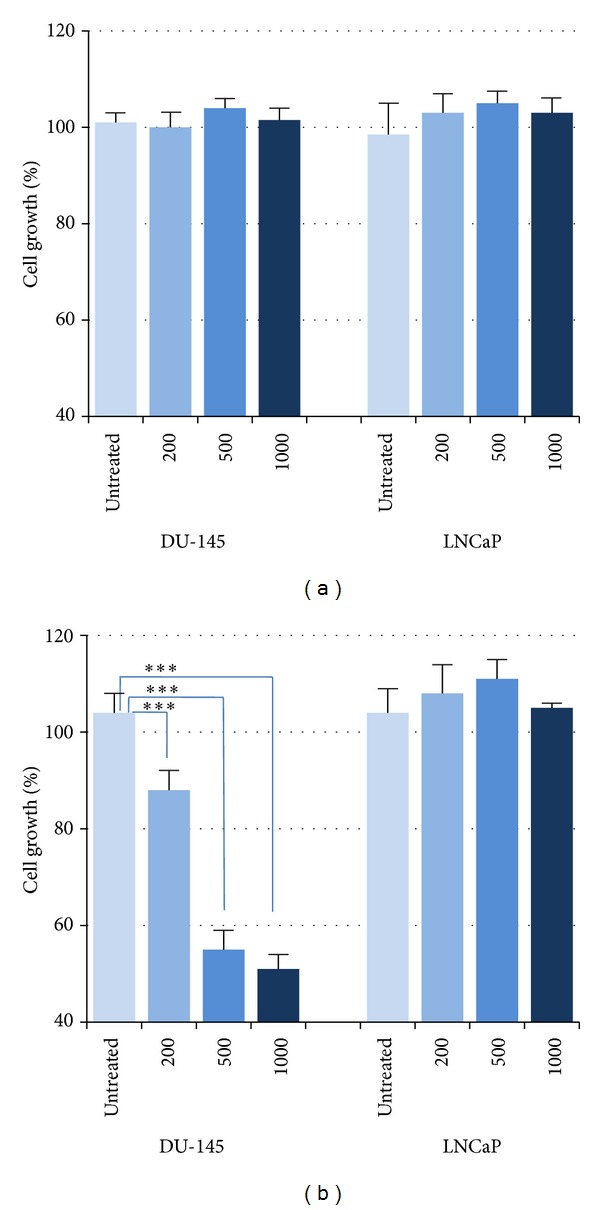
Percentages of cell growth of DU-145 and LNCaP cells after 24 h of treatment with scFv C5-I (a) and scFv C5-II (b). Treatment with scFv C5-I had no effect on the growth of the cancer cells (a). All concentrations of scFv C5-II (200, 500, and 1000 scFv/cell) significantly inhibited the cell growth of DU-145 cells with no effect on PSCA-negative LNCaP cells (***P  value <0.001).

The antiproliferative effects of different concentrations of C5-I and C5-II antibodies on DU-145 and LNCaP cells were evaluated by MTT assay. Treatment with concentrations 200, 500, and 1000 scFv C5-II showed significant antiproliferative effect on Du145 cells after 24 hrs compared to treatments with helper phage, media-treated, and untreated control conditions (*P*  value <0.05). No significant anti-proliferative effect was obtained for C5-I scFv. However, the percentages of cell growth of DU-145 cells treated with C5-II scFv were 80%, 39% and 35% for concentrations 200, 500, and 1000 scFv/cell, respectively. No inhibitory effect on the proliferation of LNCap cells was observed (Figure 4).

### 3.5. Annexin-V Assay

**Figure 5:**
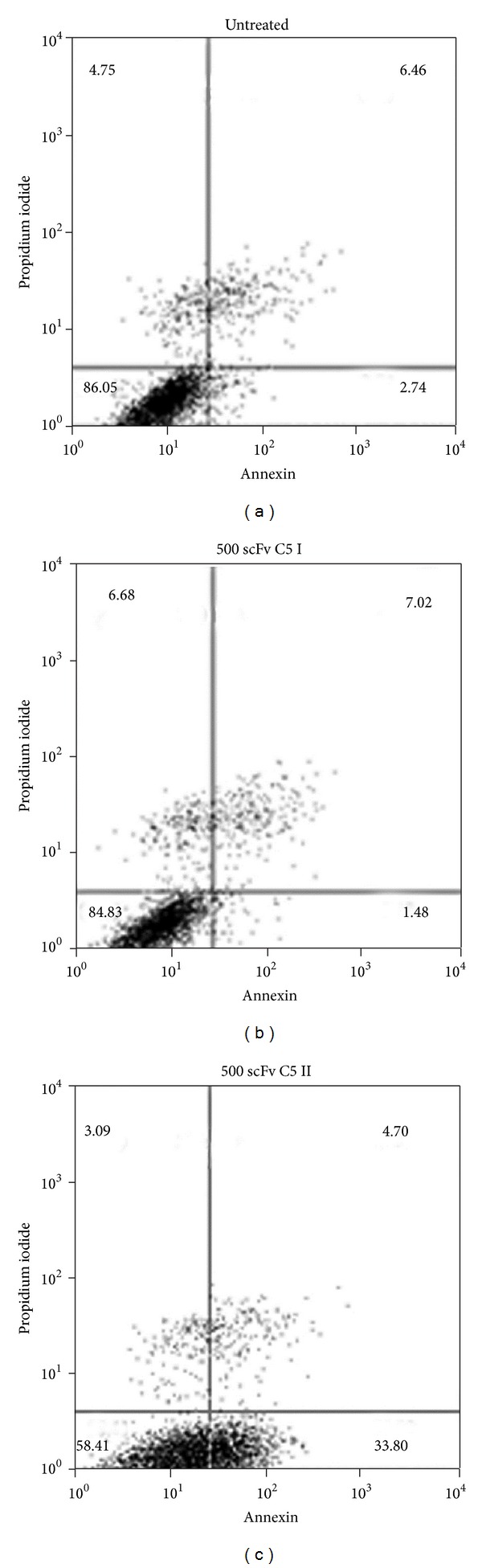
Dot plot analysis of Annexin-V/PI assay using PSCA-expressing DU145 cells. The lower left quadrant contains vital population and the lower right contains the apoptotic (AnnexinV+/PI−) population. Untreated cells (control) (a), scFv C5-I treated cells (b), and scFv C5-II treated cells (c). The scFv C5-II induced apoptosis 33.8% in DU-145 cells while the apoptotic indexes 1.48% and 2.74% were detected for scFv C5-I treated cells and untreated cells, respectively.

The results revealed that scFv C5-II significantly induced apoptosis in DU-145 cells after 24 h of treatment compared to scFv C5-I treated cells and untreated cells (33.8% versus 1.48% and 2.74%, resp.) (Figure 5).

## 4. Discussion

Prostate stem cell antigen has previously been targeted with different strategies including monoclonal antibodies and vaccines in preclinical studies [13, 27]. PSCA has been proposed as a therapeutic target for immunotherapy. Various forms of anti-PSCA antibodies have been studied including monoclonal antibodies and recombinant formats like humanized antibodies, diabodies, and minibodies that effectively targeted PSCA [28–31]. It has been reported that monoclonal antibody 1G8 which targets PSCA is able to induce cell death in vitro and prevents tumor metastasis in mice inoculated with human prostate cancer cells [32, 33]. Due to the well-known advantages of single chain antibodies over the intact monoclonal antibodies, this antibody format has been used for cancer biomarker targeting. In this study, we selected a specific and functional human single chain antibody against PSCA to be used in targeted therapy of prostate cancer. In order to select specific single chain antibodies against PSCA, two immunodominant epitopes of PSCA were used. Peptide I (TARIRAVGLLTVISK) was amino acids 50–64 of extracellular domain of PSCA molecule. It was reported that mAb 1G8 recognizes the middle portion of PSCA extracellular domain, amino acids 46–85 [33]. Therefore, peptide I used in this study is in the recognition part of 1G8 antibody which has been introduced as a biologically active fragment of PSCA molecule and is recognized by anti-PSCA antibody [34]. Peptide II (SLNCVDDSQDYYVGK) was amino acids 67–81 of PSCA protein. This peptide also has immunogenic properties and is considered as an amino acid sequence which is able to elicit the generation of antibodies [35, 36]. As examined by phage ELISA, the isolated scFv against these two fragments specifically bound to the corresponding peptides compared to control peptides. The results of cell proliferation assay indicated that the treatment of DU-145 cells with different concentrations of C5-II scFv leads to significant inhibition of cell growth in comparison with untreated cells. Growth inhibition of 61% on DU145 cells was obtained when 500 scFv/cell had been used. However, no inhibitory effect on the growth of PSCA-negative LNCaP cells was observed. Treatment of DU145 cells with scFv C5-I did not induce antiproliferative effect. In a similar study by Gu et al. [32], the effect of anti-PSCA monoclonal antibody 1G8 on the proliferation of a PSCA-transfected prostate cancer cells, LNCaP-PSCA cell line, was investigated. The antibody significantly inhibited the cell growth of LNCaP-PSCA cells, indicating the direct effect of antibody on PSCA-expressing cells. The results of Annexin-V/PI assay confirmed the MTT results and showed the mechanism of action of the scFv C5-II. The apoptotic effect 33.80% in DU145 cells treated with C5-II versus the apoptosis 2.74% in untreated cells indicated the ability of the selected scFv antibody in the induction of prostate cancer cell death. Although the anti-PSCA monoclonal antibody 1G8 was also able to induce cell death in PSCA-expressing cells [32]; the small size of the selected scFv C5-II is a great advantage which contributes to rapid tissue penetration.

Prostate cancer targeted therapy is a new approach in clinical trials and a number of studies including targeting angiogenesis, tubulin, and Bcl-2 to trigger apoptosis are being conducted [37, 38]. To develop antibodies with high specific and high-affinity properties for translation into the clinic, single chain antibodies have been developed against prostate antigens. Anti-MUC1 di-scFvs has been developed as an effective agent for targeted radioimmunotherapy (RIT) to increase the therapeutic index of RIT in epithelial cancers such as prostate tumors [39]. A scFv that binds to the prostate-specific membrane antigen (PSMA) has been also reported for its use in targeted therapeutic applications [40]. Functional single chain antibodies against PSCA would yield a new possibility for the therapy of prostate tumors since a significant level of PSCA expression has been detected in the tumors of the majority of patients with prostate cancer [3]. Manipulating the tumor-specific scFvs by genetic engineering enables the production of antitumor fusion proteins with additional effector functions. This property of scFvs has made these antibodies more applicable in therapeutics. It has been reported that an anti-PSCA scFv originated from a monoclonal anti-PSCA antibody was used in a construction of a chimeric receptor capable of inducing cytotoxicity against PSCA-positive tumor cells [41]. The specific anti-PSCA scFv C5-II selected in this study which is originated from a human antibody library and induces prostate cancer cell death offers a promising prostate cancer therapeutic agent. The results suggest a further evaluation of the scFv C5-II towards its application in the treatment of prostate cancer.
